# North-Eastern Hospital for Children

**Published:** 1902-05-24

**Authors:** 


### NORTH-EASTERN HOSPITAL FOR CHILDREN.

A special meeting of the governors of this institution will be held on Friday the 23rd instant, after we go to press, to effect the exchange of a small portion of land belonging to the hospital, so as to rectify part of the eastern boundary of the hospital property, and to borrow on mortgage of the property of the hospital, if necessary, any sum not exceeding £12,000 to enable the committee to defray the cost of the new buildings now in progress. Including the sum of £1,800 raised at the ceremony of laying the memorial stone of the new buildings on the 8th instant, the committee have in hand or in prospect nearly £19,000 of the £30,000 required. An important drawing-room meeting will be held at Lady Ampthill's, 19 Stratford Place, during the first week in June, with the object of raising additional funds.

There is probably no hospital in London which does better work than the North-Eastern Hospital for Children, and we certainly know of none where the need for new buildings is greater or the work better deserving of the liberal contributions of the public. We hope, therefore, that the committee may receive adequate support from the more intelligent of hospital givers who realise the difficulties which have to be faced in the present instance, owing to the hospital being situated in the centre of a poor district where it is hidden away from the observation of the wealthy. This hospital is entitled to receive special attention from all who desire to give wisely to those London hospitals which base their claim upon the character of the work done, for it provides relief for those members of the population only who are entitled to free hospital treatment.

